# Mechanistic elucidation of Juglanthraquinone C targeting breast Cancer: A network Pharmacology-based investigation

**DOI:** 10.1016/j.sjbs.2023.103705

**Published:** 2023-06-15

**Authors:** Hina Qayoom, Mustfa Alkhanani, Abdullah Almilaibary, Suliman A. Alsagaby, Manzoor Ahmad Mir

**Affiliations:** a Department of Bioresources, School of Biological Sciences, University of Kashmir, Srinagar 190006, India; b Department of Family and Community Medicine, Faculty of Medicine, Al Baha University, Albaha 65511, KSA; c Department of Biology, College of Science, Hafr Al Batin University of Hafr Al-Batin, 31991, KSA; d Department of Medical Laboratory Sciences, College of Applied Medical Sciences, Majmaah University, AL-Majmaah 11932, KSA

**Keywords:** Breast cancer, Juglanthraquinone C, Network pharmacology, Docking and Simulation

## Abstract

Breast cancer is the leading cause of death among women worldwide. Despite the recent treatment options like surgery, chemotherapy etc. the lethality of breast cancer is alarming. Natural compounds are considered a better treatment option against breast carcinoma because of their lower side effects and specificity in targeting important proteins involved in the aberrant activation of pathways in breast cancer. A recently discovered compound called Juglanthraquinone C, which is found in the bark of the *Juglans mandshurica* Maxim (Juglandaceae) tree has shown promising cytotoxicity in hepatocellular carcinoma. However, not much data is available on the molecular mechanisms followed by this compound. Therefore, we aimed to investigate the molecular mechanism followed by Juglanthraquinone C against breast cancer. We used the network pharmacology technique to analyse the mechanism of action of Juglanthraquinone C in breast cancer and validated our study by applying various computational tools such as UALCAN, cBioportal, TIMER, docking and simulation. The results showed the compound and breast cancer target network shared 31 common targets**.** Moreover, we observed that Juglanthraquinone C targets multiple deregulated genes in breast cancer such as TP53, TGIF1, IGF1R, SMAD3, JUN, CDC42, HBEGF, FOS and signaling pathways such as PI3K-Akt pathway, TGF-β signaling pathway, MAPK pathway and HIPPO signaling pathway**.** A docking examination revealed that the investigated drug had a high affinity for the primary target TGIF1 protein. A stable protein–ligand combination was generated by the best hit molecule, according to molecular dynamics modeling. The main aim of this study was to examine Juglanthraquinone C's significance as a prospective breast cancer treatment and to better understand the molecular mechanism this substance uses in breast cancer since there is a need to discover new therapeutics to decrease the load on current therapeutics which also are currently ineffective due to several side effects and development of drug resistance.

## Highlights

• Due to limited efficacy, side effects and development of resistance against current chemotherapy there is a need for developing targeted therapy and one such promising approach is the exploration of natural compounds through cost-effective and less time-consuming computational tools such as network pharmacology and molecular docking and simulation studies.
• On screening we found one such natural compound Juglanthraquinone C which has revealed promising prospective as an anti-cancer agent in breast carcinoma due to its ability to reduce inflammation, inhibit tumor growth, and suppress metastasis, as well as its synergistic effects with chemotherapy, which makes it a promising candidate for breast cancer treatment.
• By using the network pharmacology and other in silico approaches we for the first time revealed the potential therapeutic targets of this compound in breast carcinoma and found that the compound and breast carcinoma target network shared 31 common targets.

## Graphical abstract

**Figure:**
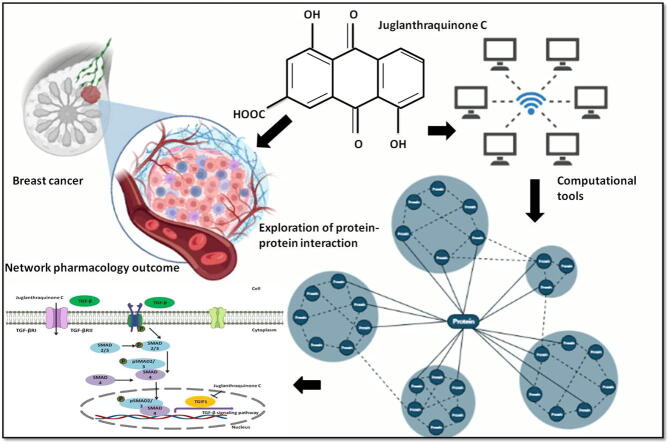

## 1 Introduction

Today, breast carcinoma accounts for one in every eight cancer diagnoses and two million new cases in people of both genders combined, overtaking lung carcinoma to be the major frequently diagnosed carcinoma globally (Sung et al., 2021). With a quarter of all female cancer cases in 2020 being diagnosed with it, undeniably it was the most prevalent kind of cancer among females and its prevalence has been rising globally, especially in transitional nations (Heer et al., 2020). Breast cancer killed an estimated 685,000 women in 2020, accounting for 16% of all cancer deaths in women. The WHO recently launched the Global Breast Cancer Initiative in response to the formerly inadequate public health response to this development (Anderson et al., 2021). If current trends continue, the mortality troll will have increased to a nerve-racking peak of 6.99 million by 2040 (Sung et al., 2021). Breast carcinoma is the most frequent malignant tumor in women in this situation and both its frequency and death rate have rapidly increased in current years. The lengthy survival rate of breast cancer patients after surgery or chemotherapy treatments is still dismal due to the substantial risks of recurrence and metastasis in this disease. The leading factor in patient fatalities is breast cancer metastases (Li et al., 2017). TNBC, the most serious kind of breast cancer is hardest to treat and more likely to spread after diagnosis (Razak et al., 2019, Qayoom et al., 2021). Therefore, there is an urgent demand for fresh natural-source treatment medications with effective and secure effects for this kind of carcinoma (Mir et al., 2020, Mehraj et al., 2022

Juglanthraquinone C, an entirely novel anthraquinone molecule that was discovered in the bark of *J. mandshurica*, is considered to have promising cytotoxicity against cancers such as hepatocellular carcinoma (Yao et al., 2012, Gao et al., 2016, Hou et al., 2016). It is common in Korea and northeast China as the rare medicinal herb *Juglans mandshurica* Maxim (Juglandaceae). This substance has been used to treat cancer in conventional medicine using its stem, roots, bark, leaves, and seeds. Numerous natural substances, including phenolics, tetralones, naphthoquinones, diarylheptanoid, and flavonoids have been discovered in these species (Juglans) in recent years (Li et al., 2003, Min et al., 2003). However, there have only been a few reports of Juglanthraquinone C pharmaceutical investigations. Furthermore, there is no information on its anti-cancer potential and associated mechanism of action. Therefore, we sought to analyze the pharmacological properties of Juglanthraquinone C as currently there is enough load on the current chemotherapeutic drugs which are rendered ineffective due to some side effects and also development of drug resistance. In our study, we explored the cytotoxic effect of Juglanthraquinone C as well as the underlying physiological mechanism. Studies have demonstrated that Juglanthraquinone C has considerable anti-tumor effects via causing S-phase arrest of the cell cycle and intrinsic apoptotic death in liver cancer, suggesting that it may be a hopeful chemotherapeutic option for the diagnosis of breast carcinoma (Lin et al., 2011). Network pharmacology-based analysis has emerged as a trustworthy way to methodically identifying the biological mechanisms underlying complicated diseases and the molecular consequences of therapeutic interventions (Zhao and Iyengar 2012). It is emerging as a possible method for the conventional medicine sector's next-generation approach to drug discovery and development since it combines information technology with systematic medicine. Network pharmacology is especially well suited for the interpretation of the mechanism of natural substances because, in contrast to traditional studies in experimental pharmacology, It concentrates on investigating multiple target control of several chemical elements (Wu and Wu, 2015, Mir et al., 2022). With the aim to forecast the underlying mechanism of action of Juglanthraquinone C (JC) and identify potentially effective components against breast cancer, we used a network pharmacological approach. Furthermore, we conducted the docking and simulation investigation to investigate and confirm the potential of JC in breast carcinoma as therapeutic.

## 2 Material and methods:

**Chemicals and Reagents**: *Juglanthraquinone C*.

The information about the compound was obtained from the literature (Yao et al., 2012, Gao et al., 2016, Hou et al., 2016). The candidate active constituent was so novel that no further information was available related to this compound even in pub chem, except a study showed its potential anti-cancer activity in hepatocellular carcinoma in *in-vitro* analysis. On the basis of the literature's existing structure, additional analysis was carried out.

### 2.1 Gene prediction for diseases and possible quantitative component

The Integrative Pharmacology-based Breast Cancer Research Platform and SWISSPROT-TARGET-PREDICTION (https://www.swisstargetprediction.ch/) were used to estimate the genes that a chemical component would affect. The Swiss target prediction approach provides information on chemical compounds and biological activities while estimating virtually all of a small molecule's macromolecular targets (Daina et al., 2019). To predict the target, the structural similarity to a known constituent was assessed. A likelihood score of less than 0.1 was established as the cutoff to screen targets from the Swiss target prediction database in order to gather favorable targets related to the compound of interest. Gene Cards (https://www.genecards.org/) which provides extensive, easy to use data on all predicted and identified genes associated with human diseases in a searchable, integrated database was used to compile the different genes linked in breast cancer (Stelzer et al., 2016). Breast cancer was used as a keyword, the key terms like “triple negative breast cancer” were also used to search the database, and targets with a gene cards inferred functionality score (GIFS) of less than 30 were chosen.

### 2.2 Screening of the common target genes

A Gene Atlas of Breast Cancer (https://t2diacod.igib.res.in/), OMIM (https://www.omim.org/) & TCGA database was used to gather the information on the common targets genes between the disease and the quantitative component (Juglanthraquinone C) (shown in Venn diagram). First, the data related to breast cancer samples with top 10% over- and under-expressed mRNA genes were collected from the Cancer Genome Atlas database. The putative targets of Juglanthraquinone C versus breast cancer genes acquired from the TCGA database were then intersected using a Venn diagram.

### 2.3 Construction of a mutual network for the disease and quantitative component

Core regulating genes can be discovered by investigating protein–protein interactions in the PPI network. The STRING database (https://stringdb.org/), which has a wealth of information about identified and anticipated protein–protein interactions of many species can be used to find PPI information (Szklarczyk et al., 2016). STRING was used to find protein–protein interactions (PPIs), with the cutoff parameter being > 0.4 (minimum confidence). In this experiment, “Homo sapiens” was the only species allowed, and before submitting the validated targets to STRING, high confidence scores > 0.7 were reserved. Data from PPI were eventually obtained. The top 30 proteins with the greatest degrees were thought to be the main targets for Juglanthraquinone C against breast cancer.

### 2.4 Using the Kyoto Encyclopedia of genes and Genomes (KEGG) and gene Ontology (GO), pathway enrichment analysis

Then, using Shiny GO, Gene Ontology analysis and KEGG pathway enrichment analysis was carried out on the shared target genes. A bubble chart and bar chart have been utilized to display the results. Only “Homo sapiens” was allowed as a species. In terms of enrichment analysis, the KEGG pathway enrichment bubble and bar charts were used to illustrate the outcome of enriched GO keywords for biological process (BP), cellular component (CC) and molecular function (MF).

### 2.5 Network analysis of interactions between compound Prescription-Active Component, disease target Gene, and pathway

Cytoscape 3.8.0 (https://cytoscape.org/.ver.3.8.0) was used to create a complex of chemical prescription-active component-disease target gene-pathway interactions. Networks of molecular interactions and biological processes may be seen using the free software Cytoscape (Zhang et al., 2021). Additionally, these networks can be combined with gene expression profiles, annotations, and other complex data and analyzed using Cytoscape. To determine each node's topological coefficients, three parameters can be determined. The terms “Degree” and “Betweenness” describes the number of edges connecting one node to another in a network. In a network, “closeness” is the opposite of the total length of the shortest routes joining any two nodes. “Node” in the network stands in for the medicine, active ingredient, ailment, target gene, and pathways. The connection between the aforementioned nodes is represented by the term “Edge”. The quality indicators were assessed according to degree, and then all of the active components in the network with degrees higher than the average were chosen for screening. Degree is the most straightforward way to describe a node's centrality in a network analysis.

### 2.6 Examining the clinical significance and validating hub gene mRNA expression

Depending on the kind of sample and stage of the malignancy, the mRNA expression of the hub genes was analysed using the malignancy Genome Atlas database in UALCAN (https://ualcan.path.uab.edu). P values of 0.05 were used to determine impact. In order to determine the amounts of hub target protein expression in healthy and breast carcinoma tissues, the Human Protein Atlas (https://www.proteinatlas.org/) was employed. Analysis of the overall survival (OS) value of hub target mRNA expression was done using the TIMER cistrome database (https://timer.cistrome.org/). A log rank P value of 0.05 was established as a significant difference. The graph shows the calculated log rank P value and the hazard ratio (HR). Using the cBioPortal programme (https://www.cbioportal.org/), it was possible to identify the genetic details and relationships between the mRNA expressions of important targets. 4135 samples of breast invasive cancer in total were examined. With the use of the Genomic Identification of Significant Targets in Cancer (GISTIC) tool, the genomic profiles of 8 hub targets were investigated.

### 2.7 Active compounds-targets docking

To confirm that the principal compound and their related expected targets are accurate. The configuration of the target protein and the proposed chemical were both found in the RCSB protein (https://www.rcsb.org) and Pub Chem databases, respectively. After loading the respective structure into Pymol 2.4.1 Software (https://pymol.org/2/), the ligands were dehydrated, hydrogenated, and separated, and using Auto Dock 4.2.6, the grid box of the respective structure was created for each target.

Juglanthraquinone C binding efficiency and correct docking validation were examined utilizing docking research on the chemical in question, we used Autodock vs. 4.2.6. After merging non-polar hydrogens, the target and receptor molecules were both saved in pdbqt format. For the docking technique, a grid box measuring 45 × 52 × 49 Å was used. The protein–ligand complex was tested by docking using the Lamarckian Genetic Algorithm (LGA). The molecular docking examination was carried out three times each with population size of about 500 each with 50 solutions, 2,500,000 evaluations and a maximum generational number of 27.

### 2.8 Simulation of molecular dynamics (MDS)

Using the Desmond 2020.1 Jorgensen et al., 1983, MD simulation experiments were conducted for the TGIF1 + Juglanthraquinone C dock complex (Shaw et al., 2010). To the salvation box of 10 Å x 10 Å x 10 Å dimension the Optimized Potential for Liquid Simulations-2005 force field was applied (Bowers et al., Jorgensen et al., 1983, Chow et al., 2008, Shivakumar et al., 2010). To counteract the 0.15 M charge, sodium ions were introduced to the system, and sodium chloride solutions were injected to simulate physiological circumstances. A constant volume-constant temperature (NVT) ensemble for 10 ns was set up and then retrained over the protein ligand complexes. It was utilized to finish the rapid 12 ns equilibration and reduction procedure after the initial phase. The constant temperature-constant pressure (NPT) ensemble was created using the Nose-Hoover chain coupling approach with a relaxation time of 1.0 ps, the pressure held constant at 1 bar during each simulation and the temperature adjusted to 27 °C. (Martyna et al., 1994). The particle mesh Ewald approach was used to calculate the extended electrostatic interactions, with the coulomb association radius of 9. The bonding strengths were predicted using the RESPA integrator for each trajectory with a time step of 2 fs (Shivakumar et al., 2010). The final simulation run for the TGIF1 + Juglanthraquinone C complex was lasted to around 100 ns. For the purpose of tracking the steadiness of the MD simulations, radius of gyration (Rg), root mean square fluctuation (RMSF), the root mean square deviation (RMSD), and number of hydrogen (H-bonds) were calculated (Toukmaji and Board Jr 1996).

### 2.9 Analysis of binding free energy

The generalized Born surface area approach in conjunction with molecular mechanics was utilized to get the binding free energy values for the compound-protein complex. The mmgbsa.py Python script was used to calculate the MM-GBSA binding free energy over the duration of 50 test frames using the VSGB solvation model and the OPLS5 force field. Using the additivity principle, the binding free energy value of MM-GBSA (kcal/mol) was calculated, which entails combining several energy modules such as covalent, columbic, solvation of ligand and protein hydrogen bonds, van der Waals, self-contact and lipophilic. The algorithm shown below was used to determine the value of “ΔGbind”:

$${\Delta G}_{\mathrm{bind}}={\Delta G}_{\mathrm{MM}}+{\Delta G}_{\mathrm{Solv}}-{\Delta G}_{\mathrm{SA}}$$

Where.

• ΔG_SA_ stands for the change in the thermodynamic stability of the system when the protein and ligand come together to form a complex in a solvent environment. A negative ΔGSA indicates that the complex formation is energetically favorable, while a positive ΔGSA indicates that the complex formation is unfavorable
• ΔG_Solv_ stands for the symbol used to represent the variation of the solvent–solute interaction free energies among both the bound state and unbound state of a ligand-receptor complex. It reflects the energy required to transfer the solute molecules from the vast solvent to the bound state, compared to the energy required to relocate the individual components (the ligand and receptor) from the bulk solvent to their unbound states
• ΔGbind represents the variation in Gibbs free energy upon binding of a ligand to a receptor or enzyme. It is a thermodynamic quantity that reflects the strength of the interaction between the ligand and the receptor/enzyme, and can be used to predict the affinity of the ligand for the receptor.
• ΔGMM represents the “molecular mechanics free energy difference” and it represents the diversity in free energy between two different conformations of a molecule or a complex, calculated using molecular mechanics force fields

## 3 Results:

In this research, the compound Juglanthraquinone C was selected from literature. The properties of the selected component are summarized in Table 1. The discovered chemical compound is mentioned here as a potential molecule for more network pharmacology study, in-silico docking and MD simulation analysis.

**Table 1:** Information for Quantitative analysis of Juglanthraquinone C.

**Compound Name**	**IUPAC NAME**	**Chemical Structure**	**Reference**
Juglanthraquinone C	(1,5-dihydroxy-9,10- anthraquinone-3-carboxylic acid, JC)		(Yao et al., 2012, Gao et al., 2016, Hou et al., 2016)

## 4 Network pharmacology Analysis:

### 4.1 Construction of an interaction network and target gene screening

**Fig. 1:**
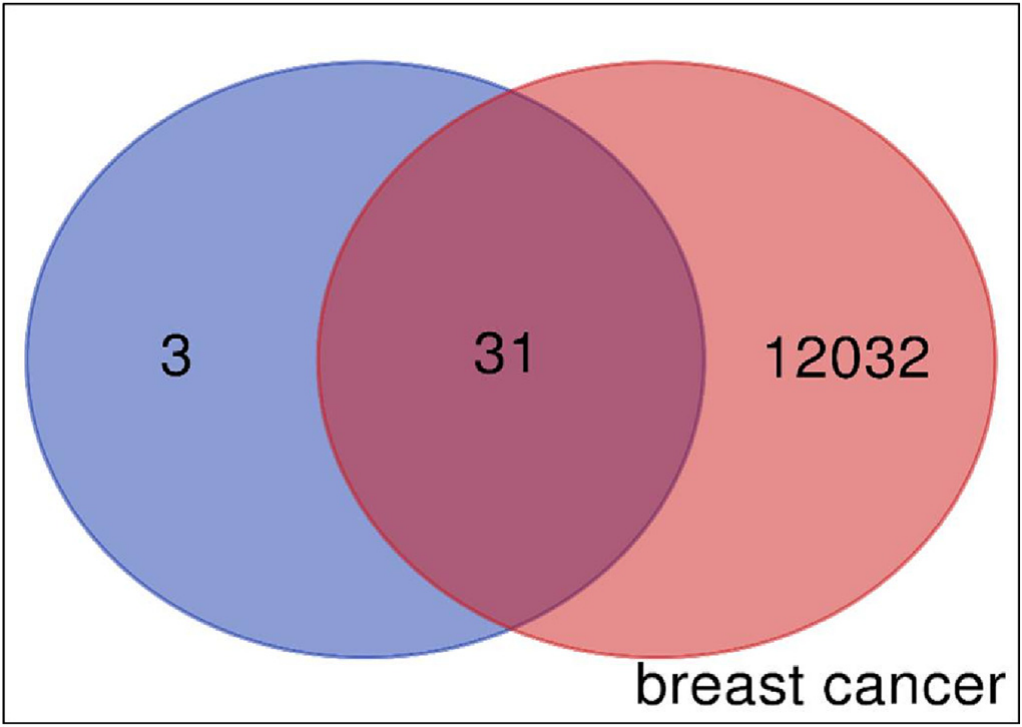
A Venn image depicts frequent target genes for compound medication and disease. The blue circle represents the target genes of Juglanthraquinone C, the red circle represents the target genes of breast cancer, and the coincident portion shows the shared target genes. The height indicates the number of target genes.

**Fig. 2:**
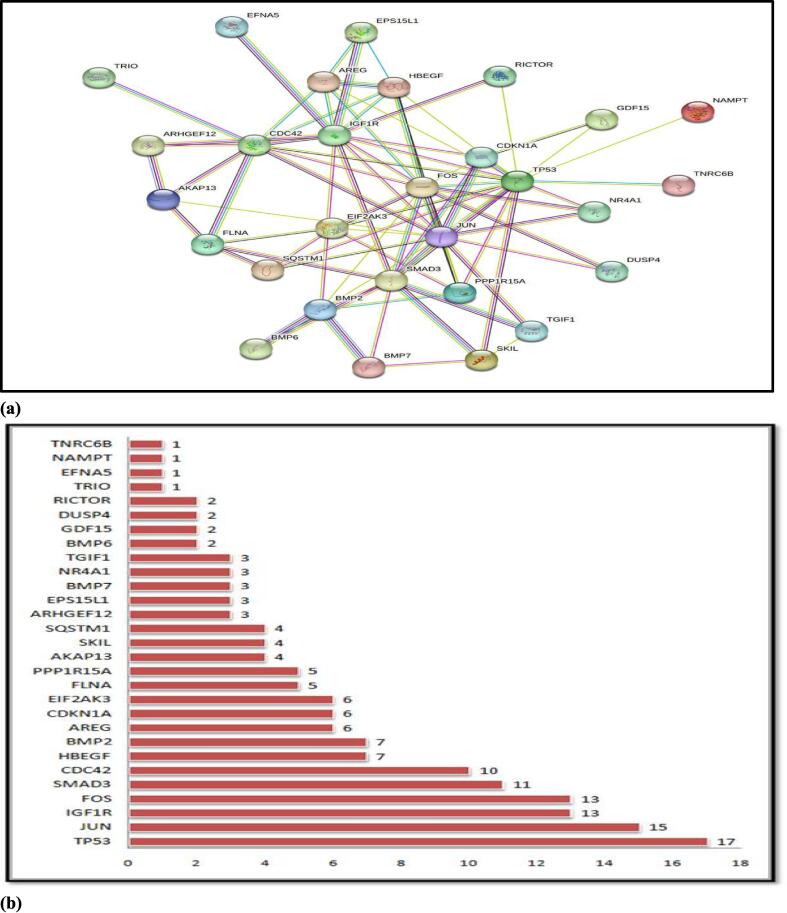
The result of the interplay between networks of shared target genes. (a) The Protein-Protein Interaction network of the common target genes. The edges depict relationships between target genes, the nodes depict target genes, the stuffing of the nodes depicts the 3D structure of the target genes, and the edges' colors depict various types of interactions, including cyan and purple for known interactions, green, red, and blue purple for predicted interactions, and chartreuse, black, and light blue for others. (b) occurrence of the top 30 common target genes.

For Juglanthraquinone C, a total of 34 putative target genes were found (Shown in Fig. 1) Supplementary File 1. Meanwhile, using Gene Cards platforms, 12,063 disease target genes linked to breast carcinoma were extracted. The quantitative compound and breast cancer both had 31 shared similar target genes. The compound Juglanthraquinone C was chosen for more examination. According to the PPI diagram for common target genes (Fig. 2**(a)**), PPI contained 31 nodes and 80 edges. Fig. 2 displays the incidence of the occurrence of the top 30 shared target genes (b). The target genes TP53, JUN, IGF1R, FOS, SMAD3, CDC42, HBEGF, BMP2, AREG, and others showed a high incidence of protein interaction, which may indicate that they are the network's node proteins Supplementary File 2 and 3. The findings demonstrated that the chosen component had a strong affinity for them and could be used as possible gene targets for the treatment of breast carcinoma.

### 4.2 Screening of key pathways of Juglanthraquinone C for treating breast carcinoma

**Fig. 3:**
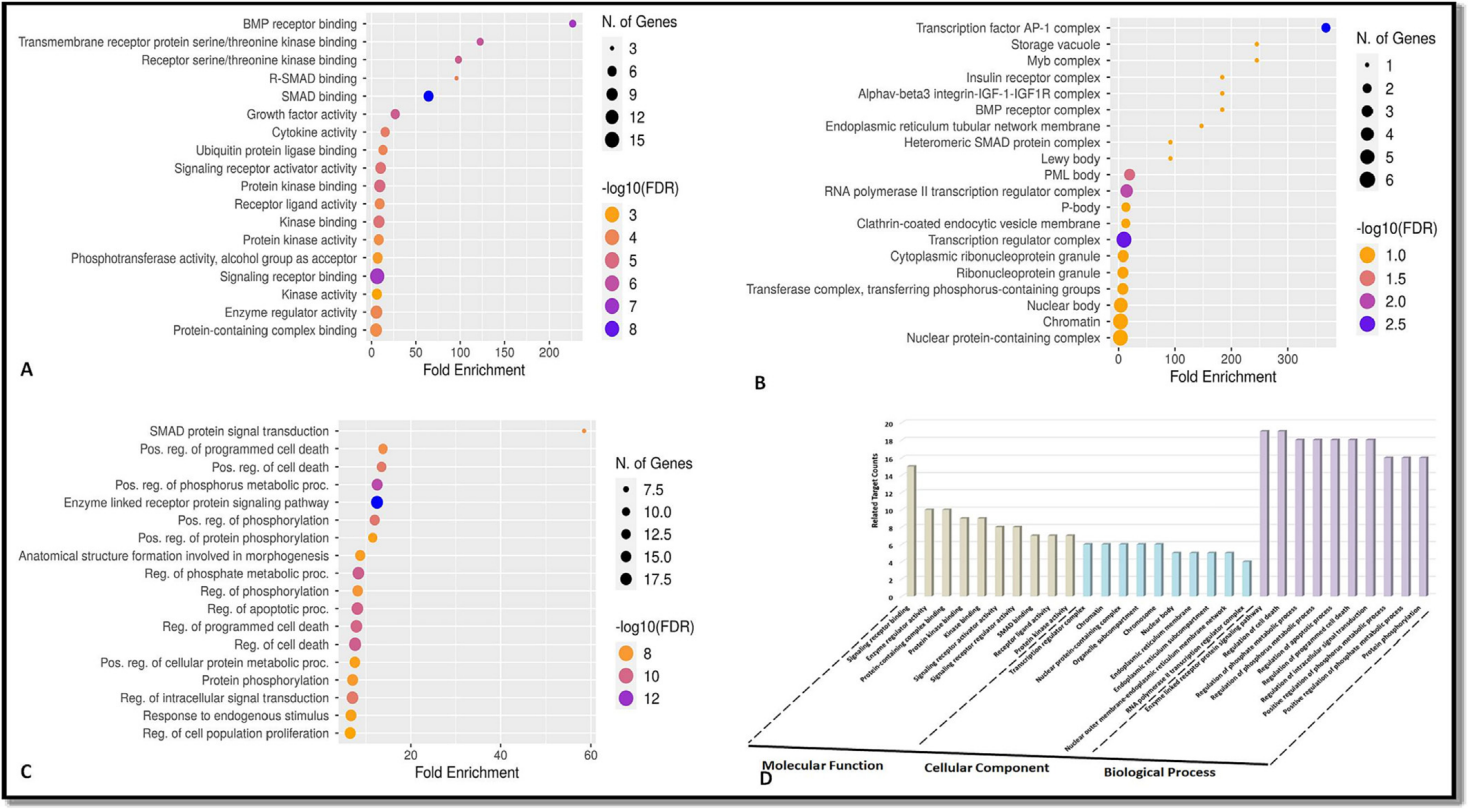
GO (Molecular function, Cellular component and Biological process) analysis (top 20): A) Gene Ontology enrichment analysis bubble chart for the term in the molecular function category. B) A bubble diagram for the Gene Ontology enrichment analysis term for the category of cellular components. C) A bubble diagram for the Gene Ontology enrichment analysis term for the biological processes category. D) The top processes determined by the numbers of associated target genes and the GO category findings.

**Fig. 4:**
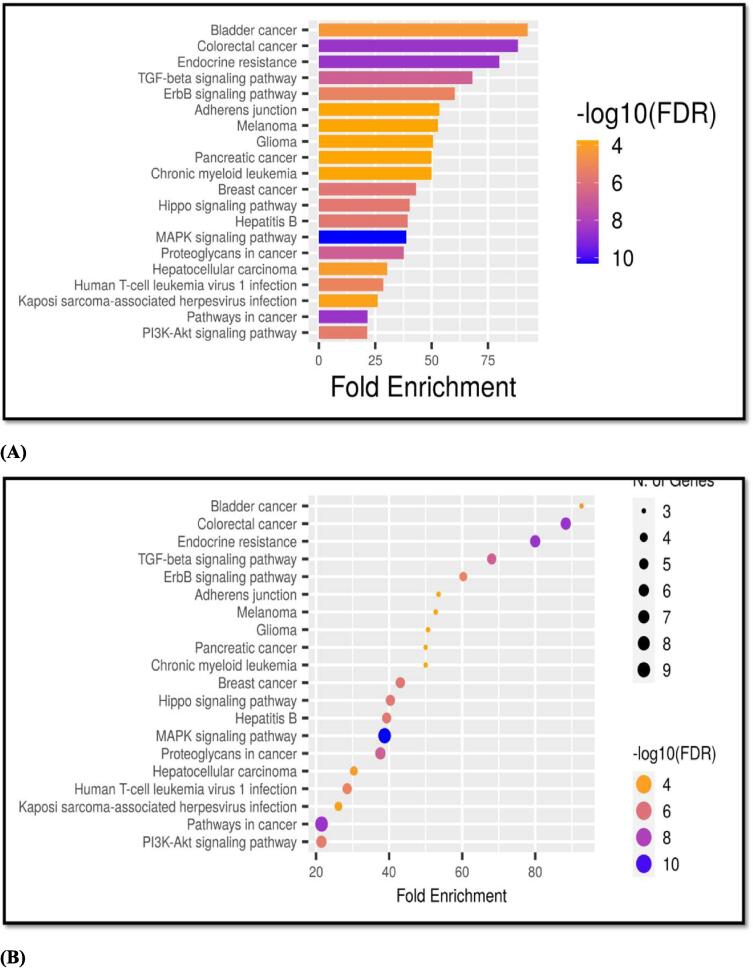
KEGG pathway enrichment analysis (top 20) (A) Bar Plot for Pathway enrichment Analysis (B) Bubble chart for Pathway enrichment Analysis.

Gene Ontology investigation of the shared target genes revealed that the biological process was mostly concerned in the control of cell death and the enzyme associated receptor protein signaling pathway (Fig. 3) Supplementary File 4, 5, 6 and 7. The primary molecular processes involved in receptor binding during signaling. Chromatin pathways and the transcription regulatory complex were seen in the cellular component (Fig. 4**)** displays the enrichment analysis (KEGG) of the abovementioned frequent target genes. The top 20 signaling pathways were chosen after wide routes were eliminated, and they are enlisted in Table 2 Supplementary File 8. This indicated the outcome of Juglanthraquinone C for breast carcinoma diagnosis may operate on many diverse pathways and involve intricate interactions between these pathways.

**Table 2:** Analysis of Pathway Enrichment (top 20).

**Signaling Pathway**	**nGenes**	**Fold Enrichment**	**Enrichment FDR**
Bladder carcinoma	3	92.66667	4.19E-05
Colorectal cancer	6	88.35659	2.51E-09
Endocrine resistance	6	79.98596	2.51E-09
TGF-beta signaling pathway	5	68.08841	1.75E-07
ErbB signaling pathway	4	60.30688	5.78E-06
Adherens junction	3	53.51174	0.00017659
Melanoma	3	52.76852	0.00017659
Glioma	3	50.65778	0.00017659
Pancreatic cancer	3	49.99123	0.00017659
Chronic myeloid leukemia	3	49.99123	0.00017659
Ovarian steroidogenesis	2	49.66449	0.002101342
Endometrial cancer	2	43.6705	0.002542103
Breast cancer	5	43.07634	1.50E-06
Hippo signaling pathway	5	40.33263	1.82E-06
Basal cell carcinoma	2	40.20459	0.002934028
Hepatitis B	5	39.33057	1.84E-06
Prostate cancer	3	39.16838	0.000349086
Inflammatory bowel disease	2	38.96752	0.003058191
MAPK signaling pathway	9	38.76871	4.83E-11

### 4.3 Network of Prescription-Active components in a compound and disease targets

**Fig. 5:**
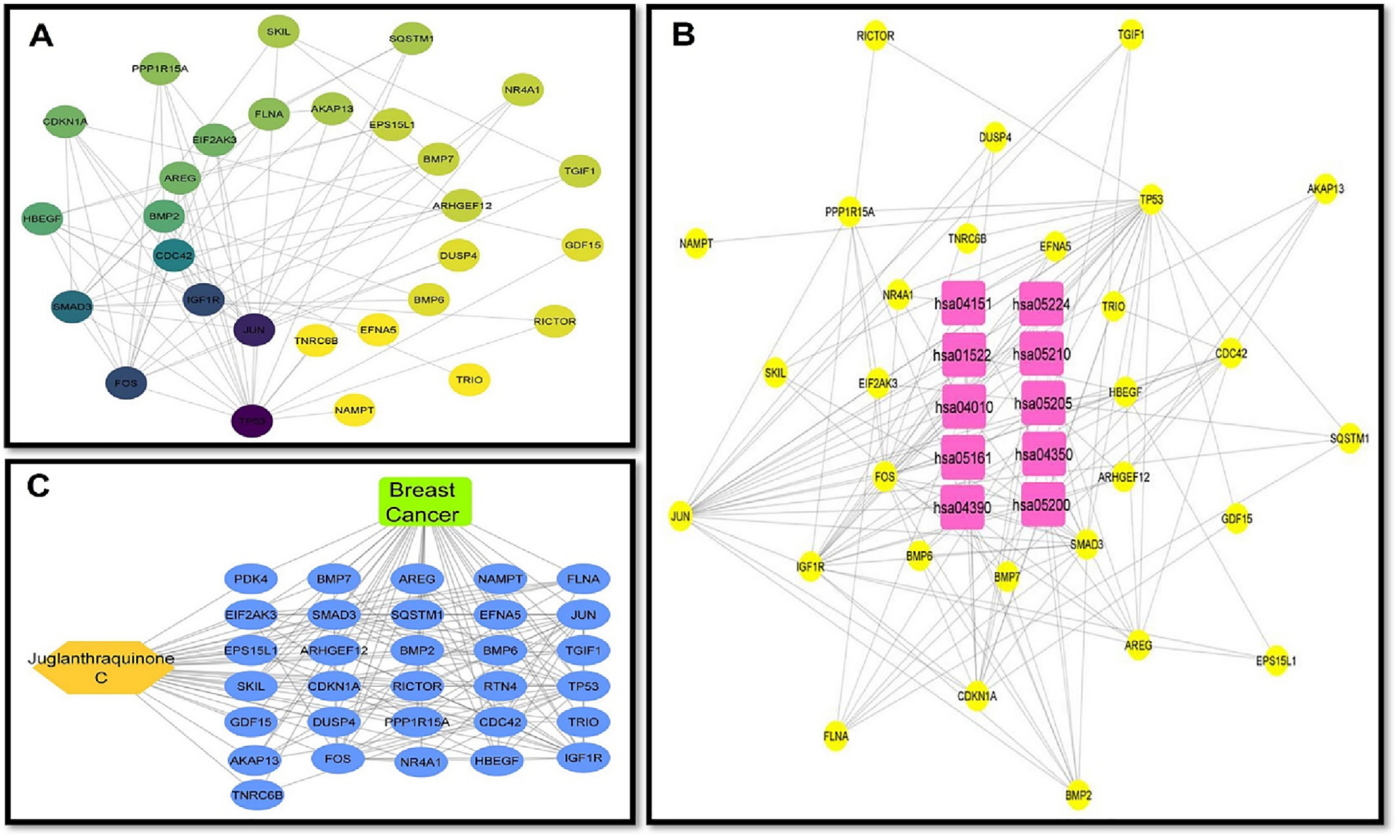
A) A network of protein–protein interactions based on shared targets. B) The key KEGG pathway network derived from KEGG enrichment analysis. C) The 31 frequent targets (presented as blue oval nodes), the Juglanthraquinone C (shown as orange hexagon node).

Fig. 5 displays the discovery of the prescription-active compound and disease targets and pathway interaction complex. There were 35 nodes in the network (34 target genes and 1 active component). Additionally, Table 3 displays the interaction network outcomes for this active chemical. Juglanthraquinone C has a degree of 31. The aforementioned findings demonstrate that quality markers may affect the entire biological network system as opposed to just a single target gene.

**Table 3:** Interaction network details of Juglanthraquinone C.

**Component**	**Degree**	**Target gene**
Juglanthraquinone C	31	*AKAP13, AREG, ARHGEF12, BMP2, BMP6, BMP7, CDC42, CDKN1A, DUSP4, EFNA5, EIF2AK3, EPS15L1, FLNA, FOS, GDF15, HBEGF, IGF1R, JUN, NAMPT, NR4A1, PDK4, PPP1R15A, RICTOR, RTN4, SKIL, SMAD3, SQSTM1, TGIF1, TNRC6B, TP53, TRIO*

The Cytoscape tool was utilized to import the Protein-protein Interaction data from the STRING platform and generate a Protein-Protein Interaction network based on shared targets, as shown in network Fig. 5**A**. The protein nodes are organized in degrees with yellow color representing higher and blue representing lower degree of occurrence. Based on KEGG enrichment analysis, the KEGG major pathway network is shown in Fig. 5**B** and Table 4. Juglanthraquinone C is shown in Fig. 5**C** as a yellow hexagonal node, along with 31 common targets and the edges connecting them.

**Table 4:** Top 10 signaling pathways according to gene ratio & the genes they interact with.

**Pathway Id**	**Pathway**	**Target genes**
**hsa04151**	PI3K-Akt pathway	AREG NR4A1 CDKN1A IGF1R TP53 EFNA5
**hsa05224**	Breast cancer	CDKN1A IGF1R TP53 FOS JUN
**hsa01522**	Endocrine resistance	HBEGF CDKN1A IGF1R TP53 FOS JUN
**hsa05210**	Colorectal cancer	AREG CDKN1A TP53 SMAD3 FOS JUN
**hsa04010**	MAPK signaling pathway	AREG DUSP4 NR4A1 IGF1R TP53 FOS JUN EFNA5 FLNA
**hsa05205**	Proteoglycans in cancer	HBEGF CDKN1A IGF1R TP53 ARHGEF12 FLNA
**hsa05161**	Hepatitis B	CDKN1A TP53 SMAD3 FOS JUN
**hsa04350**	TGF-β signaling pathway	BMP7 BMP2 BMP6 SMAD3 TGIF1
**hsa04390**	HIPPO signalling pathway	BMP7 AREG BMP2 BMP6 SMAD3
**hsa05200**	Pathways in cancer	CDC42 CDKN1A BMP2 IGF1R TP53 SMAD3 FOS JUN ARHGEF12

### 4.4 Validation of hub gene mRNA expression and evaluation of clinical relevance

**Fig. 6:**
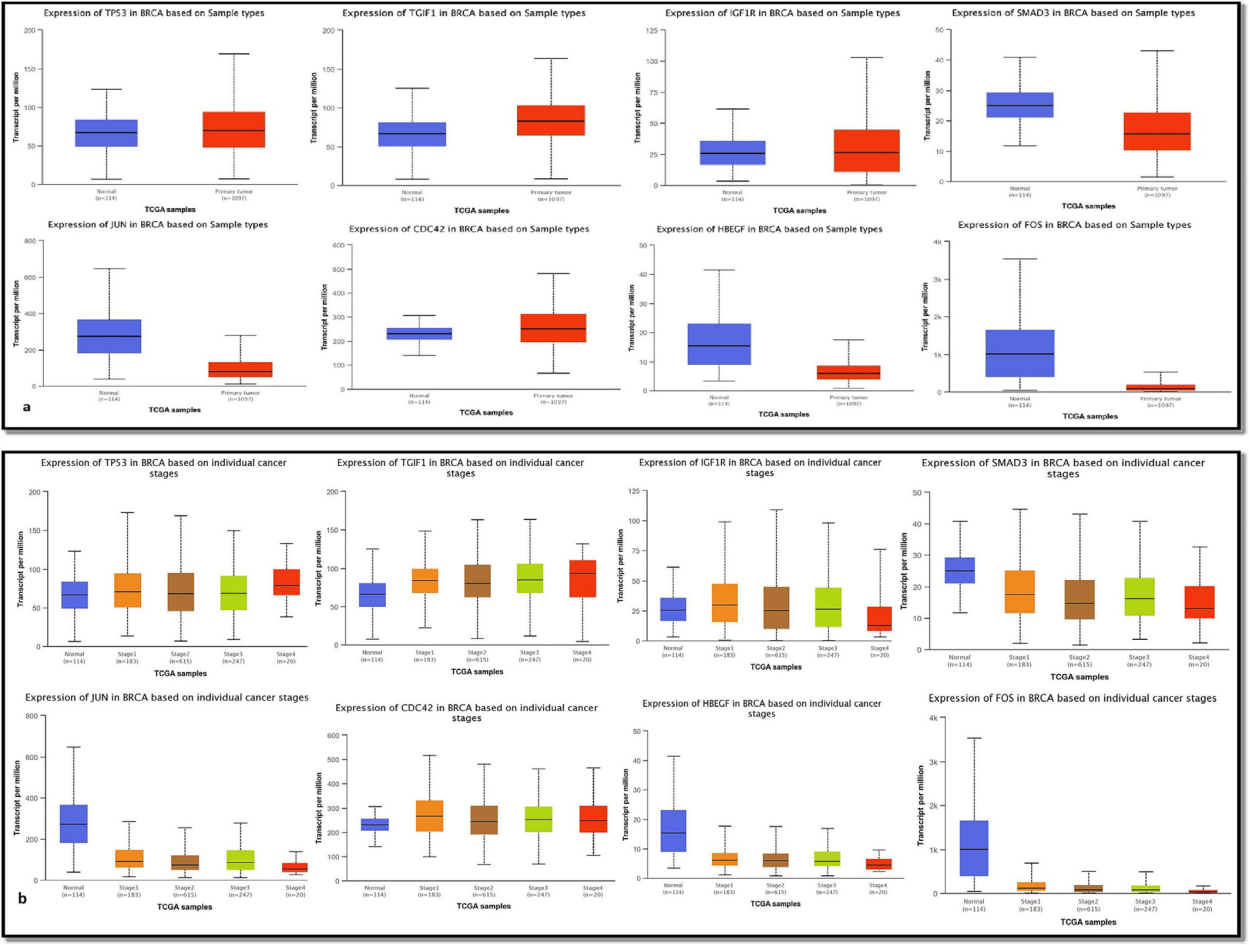
Verification of the hub targets' UALCAN mRNA expression. (a) Eight hub targets' mRNA levels in normal and BC tissues (***P0.001). (b) The mRNA levels of 8 hub targets at various phases of BC cancer development (*P less than 0.05; **P less than 0.01; ***P less than 0.001).

We looked at the mRNA and protein expression of the 8 hub targets to clarify their potential therapeutic significance and prognostic value. TGIF1, TP53 and CDC42 had significantly higher mRNA levels in tumor tissues in the invasive breast cancer patients from “The Cancer Genome Atlas database” in UALCAN with lower mRNA levels of SMAD3 and FOS that are tumor suppressors Fig. 6**a**. In turn, JUN and HBEGF mRNA expression levels were significantly reduced in tumor tissues, but IGF1R mRNA expression was not significantly reduced.

Then, we assessed the correlation between data from 8 hub targets and various BC tumor stages using the UALCAN database. The expression levels of TP53 and TGIF1 were appreciably higher than normal levels, with the exception of CDC42 which was insignificantly higher in stage four BC tissues, as shown in Fig. 6**b**. In contrast, the mRNA levels of JUN, SMAD3, FOS, HBEGF and IGF1R were significantly decreased when compared to normal levels in BC tissues in stages one through four.

**Fig. 7:**
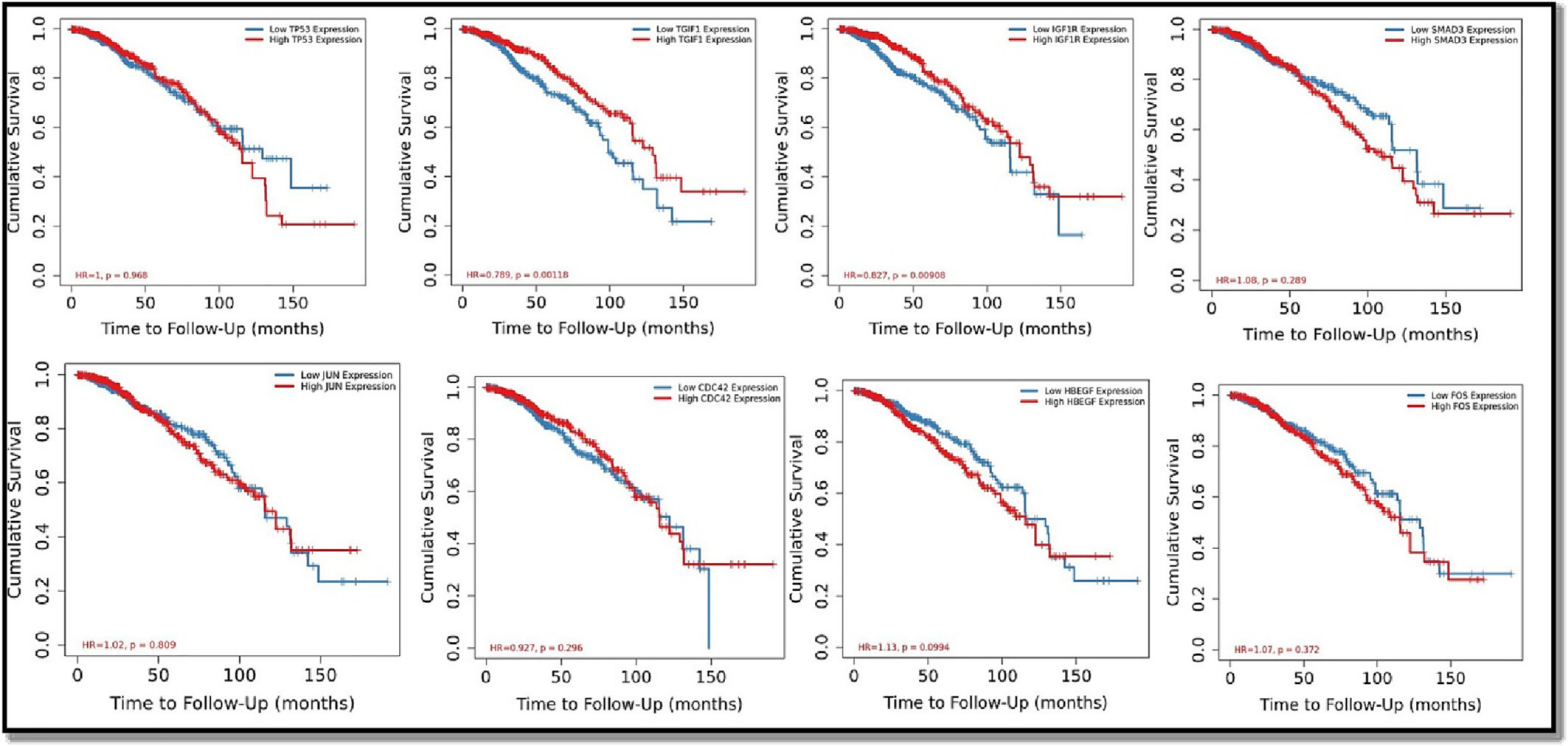
Overall Survival analysis of 8 hub targets in Breast Cancer (K-M plot).

Following that, we looked at the predictive value of 8 hub targets in breast cancer patients using the TIMER Cistrome database. According to Fig. 7, among the 8 genes, OS was significantly correlated with TP53 (hazard ratio [HR] = 1, log-rank P = 0.968), TGIF1 (HR = 0.789, log-rank P = 0.001), and IGF1R (HR = 0.827, log-rank P = 0.009). SMAD3 (HR = 1.08, log-rank P = 0.289), JUN (HR = 1.02, log-rank P = 0.809), CDC42 (HR = 0.927, log-rank P = 0.296), HBEGF (HR = 1.13, log-rank P = 0.09) and FOS (HR = 1.07, log-rank P = 0.372). The findings showed that significantly lower survival was related with significantly higher expression of SMAD3, FOS and HBEGF and low expression of CDC42, TGIF1 and IGF1R.

**Fig. 8:**
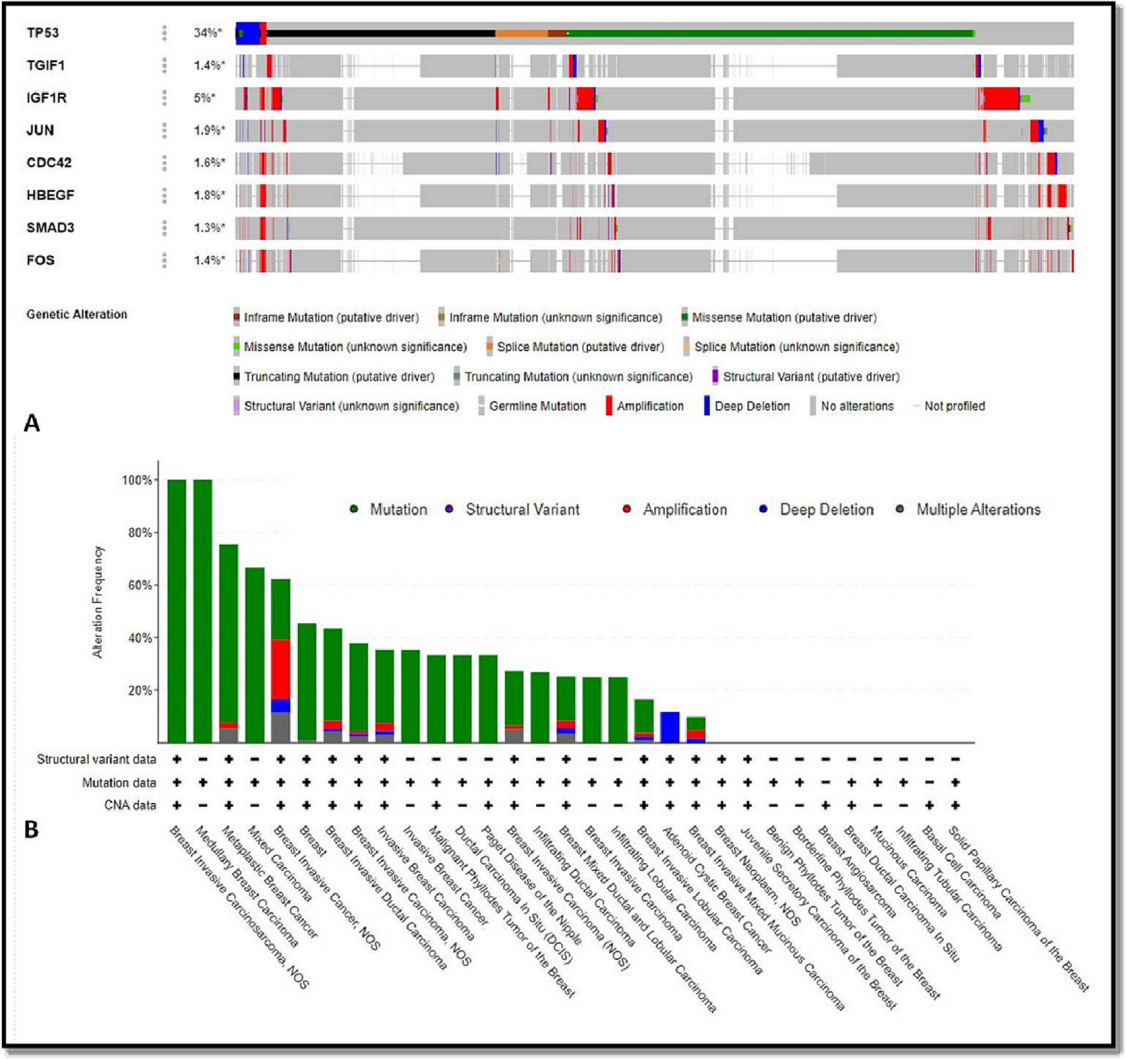
Genetic changes in eight hub targets in BC patients (cBioPortal). (a) Visual OncoPrint overview of genomic changes found in 8 hub targets. (b) A outline of changes in 8 hub targets across various BC types.

The cBioPortal programme was then used to examine the prevalence and kinds of gene alterations in 8 hub targets across 4135 breast cancer patients. According to Fig. 8**a**, the total rate of change of 8 hub targets was 38% and the individual gene alteration rates ranged from 1.3 to 34%, with TP53 having the highest rate of variation (34%) and SMAD3 having the lowest rate (1.3%). Additionally, the genetic modification rate varied from 0 to 100% across the different BC types Fig. 8**b**.

### 4.5 Molecular docking studies

**Fig. 9:**
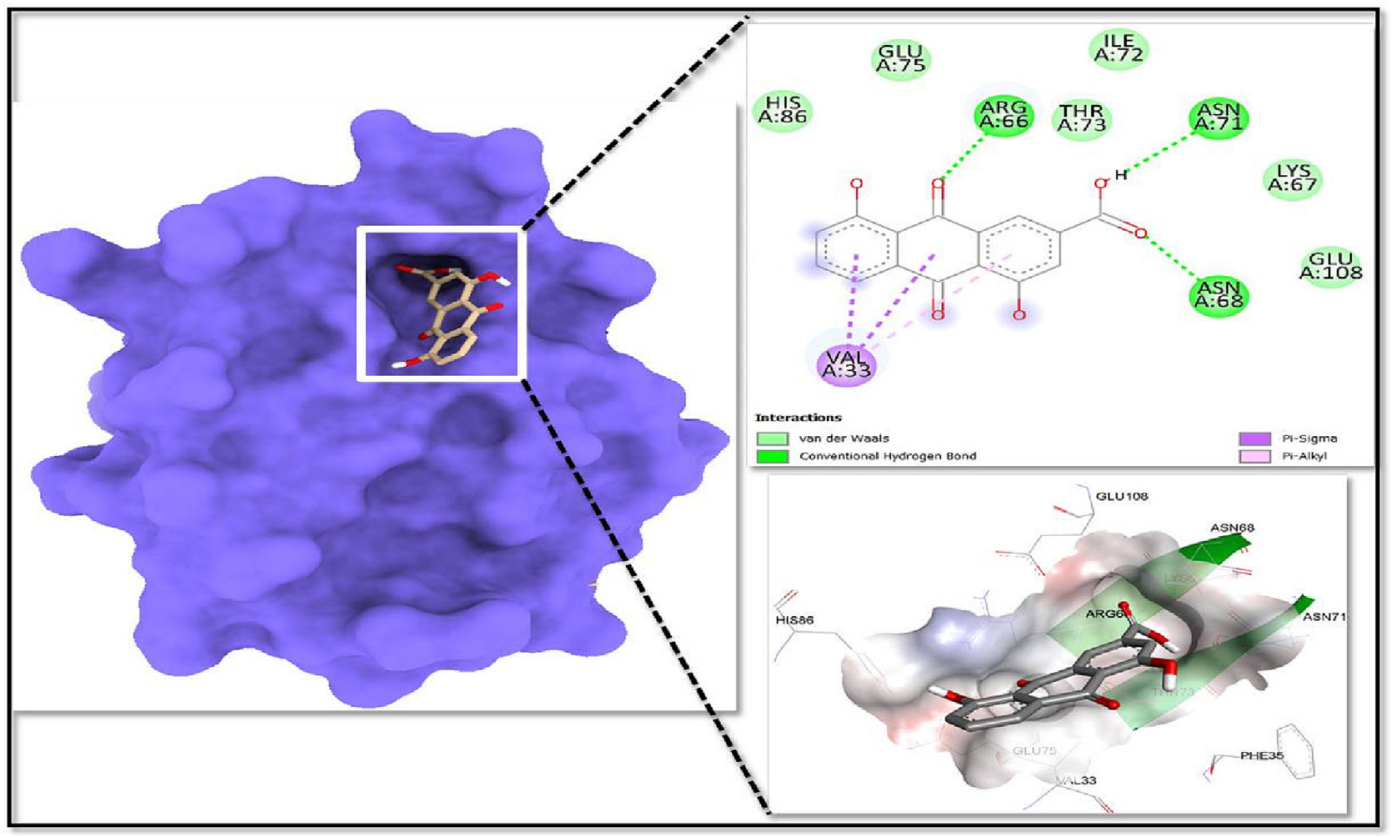
Molecular surface image of TGIF1 showing a deep cavity where Juglanthraquinone C is attached. Right upper image of the 2D interaction shows interactions between the ligand and protein, while the highly charged central ligand-bound site is visible on the right down charged surface.

All binding energy values were computed for the best cluster (95%) with the lowest Root Mean Square Deviation of 0.25. Juglanthraquinone C has shown a high binding affinity for the TGIF1 with the lowly binding energy (G −9.9 kcal/mol). Juglanthraquinone C established conventional hydrogen bonds with Arg66, Asn68, and Asn71 residues after its interaction with TGIF1 (Fig. 9), while pi-sigma and pi-alkyl contacts were formed with Val33 (Fig. 9). The interaction between the ligand Juglanthraquinone C and the deep saddle-shaped core was shown in the molecular surface image (Fig. 9). The ligand Juglanthraquinone C was seen interacting with the charged amino acid residues in the binding pocket in the charged surface view.

### 4.6 Studies using simulations for molecular dynamics

**Fig. 10:**
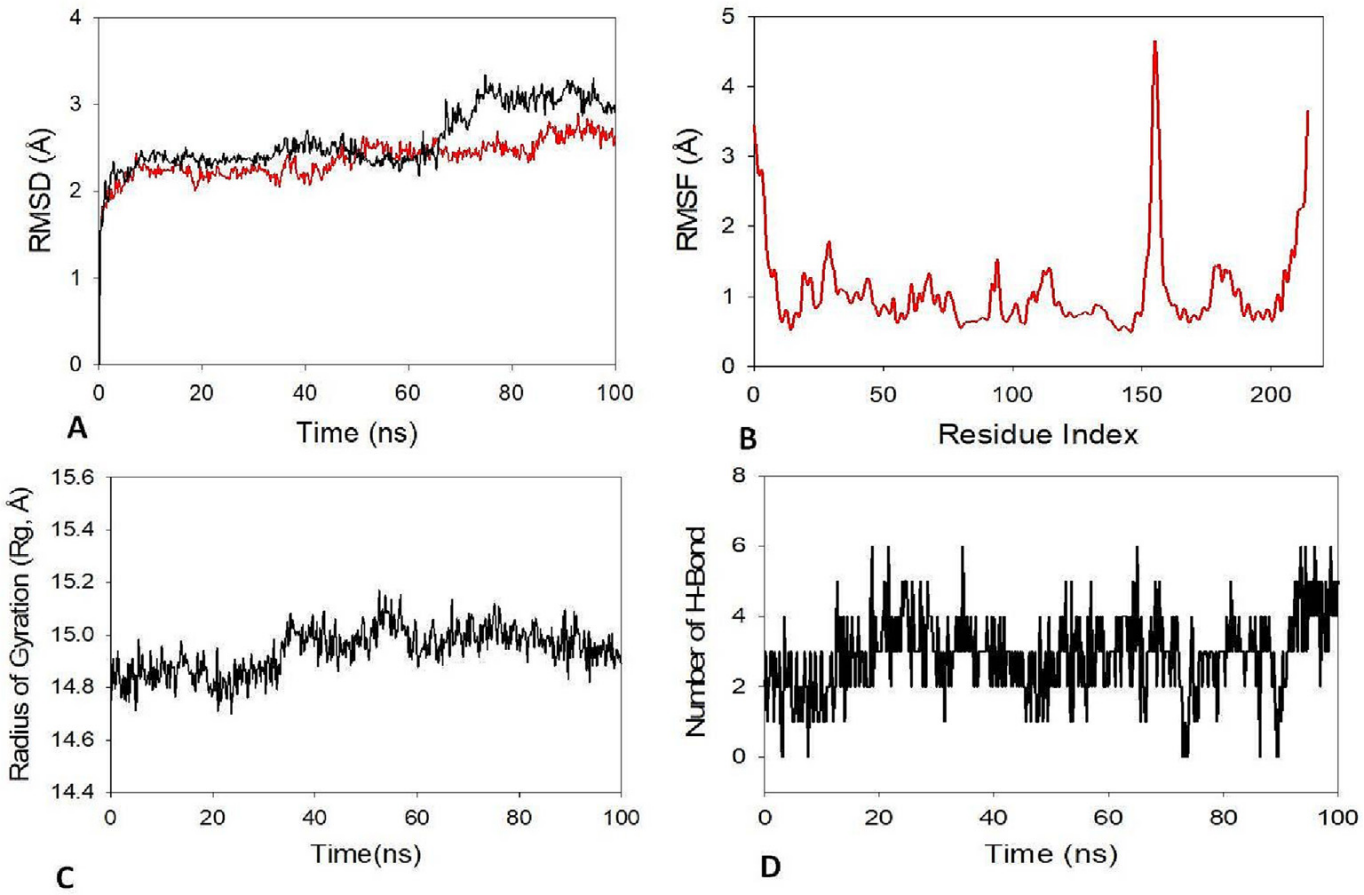
Molecular Dynaics and Simulation study of 100 ns trajectories of (A) TGIF1 C backbone RMSD (red) and RMSD of Juglan (black); (B) RMSF of TGIF1 Cα backbone; (C) Radius of gyration (Rg) of TGIF1 + Juglanthraquinone C; and (D) Formation of hydrogen bonds in TGIF1 + Juglanthraquinone C.

Molecular dynamics and simulation (MD) methods were used to examine the stability and convergence of TGIF1 + Juglanthraquinone C. When analyzing the results of the Root Mean Square Deviation, the simulation of 100 ns indicated stable conformation. When TGIF1 was bound to Juglanthraquinone C, the RMSD of the C-backbone showed a deviation of 2.3, whereas the RMSD of the ligand Juglanthraquinone C was found to be 2.8 (Fig. 10**A**). All of the RMSD values were less than 3, which is the desired range. Stable Root Mean Square Deviation plots demonstrated good convergence and stable conformations throughout the experiment. It was hypothesized that Juglanthraquinone C in complex with TGIF1 is extremely stable in complex due to the ligand's increased affinity. Juglanthraquinone C caused only minor spikes of variation in the TGIF1 protein, which may be attributable to the residues' greater flexibility, to be visible on the graph of root mean square fluctuations (RMSF) (Fig. 10**B**). The structure of the protein was hence stiff during simulation in compound-bound conformations, as determined from RMSF graphs. The protein's radius of gyration (Rg) serves as a measure of how compact it is. In this study, the TGIF1 C-backbone connected to Juglanthraquinone C maintained a consistent radius of gyration (Rg) between 14.9 and 14.8 (Fig. 10**C**). When a protein was bound to a ligand, significant stable gyration (Rg) showed that the protein is orientated in a very compact way. The number of hydrogen bonds indicated a strong and steady complex interaction between the protein and the ligand. There were four different hydrogen bonds between TGIF1 and Juglanthraquinone C during the simulation, which lasted 100 ns. (Fig. 10**D**).

### 4.7 Generalized Born surface area (MM-GBSA) computations in molecular mechanics

TGIF1 + Juglanthraquinone C binding free energy and additional contributing energy in the form of MM-GBSA were determined using the MD simulation trajectory. According to the results (Table 5), ΔGbindCoulomb, ΔGbindvdW, and ΔGbindLipo contributed most to the stability of the simulated complexes, whereas ΔGbindCovalent and ΔGbindSolvGB contributed to the instability of the comparable complexes. The binding free energies of the TGIF1 + Juglanthraquinone C complex were noticeably greater. These findings validated the promise of Juglanthraquinone C since it demonstrated greater affinity for the chosen protein and the capacity to assemble stable protein–ligand complexes.

**Table 5:** Binding free energy components for the TGIF1 + Juglanthraquinone C calculated by MM-GBSA.

**Energies (kcal/mol)**	**TGIF1 + Juglanthraquinone C**
**ΔG_bind_**	−54.85 ± 7.0
**ΔG_bind_Lipo**	−16.18 ± 1.0
**ΔG_bind_vdW**	−36.19 ± 2.0
**ΔG_bind_Coulomb**	−12.27 ± 6.1
**ΔG_bind_H_bond_**	−1.93 ± 0.3
**ΔG_bind_SolvGB**	22.34 ± 3.0
**ΔG_bind_Covalent**	7.83 ± 4.0

### 4.8 Principal component analysis

**Fig. 11:**
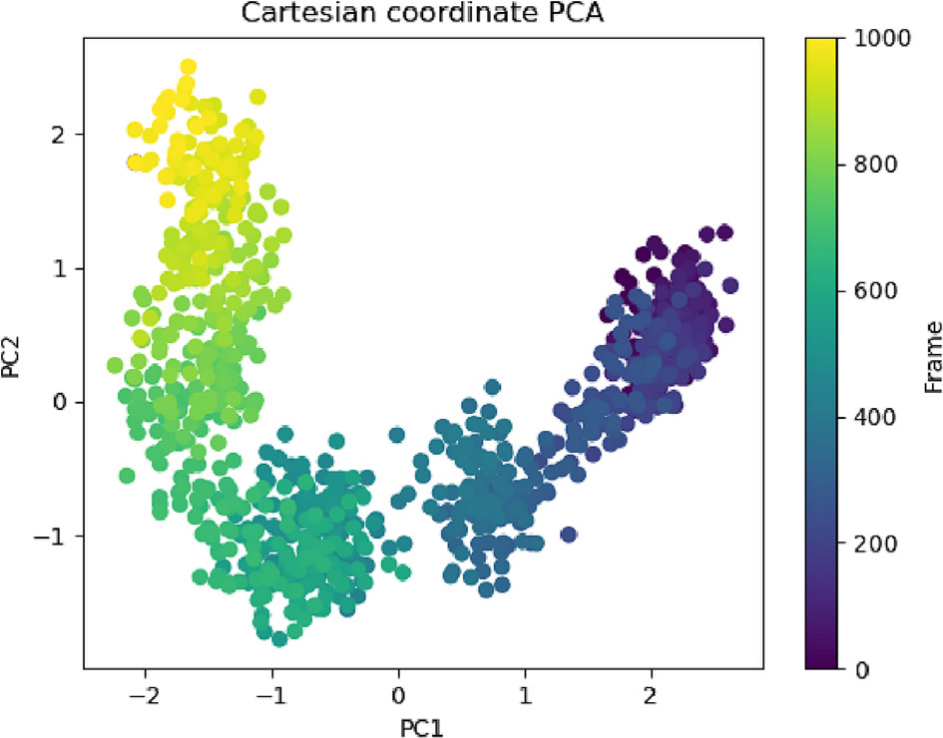
PCA analysis of Eigen values of 1000 frame Cartesian coordinates from MD trajectory for 100 ns of protein–ligand complex.

The Principal Component Analysis (PCA) technique is employed to rigorously scrutinize the molecular dynamics (MD) simulation trajectories of the protein–ligand complex. The objective of this examination is to interpret the random, global atomic displacements within the amino acid residues, as depicted in Fig. 11. The trajectories, characterized by variability and flexibility, are primarily a result of the inherent stochasticity and globally non-correlated motion integral to the protein's architecture. A covariance matrix documents the mobility of internal coordinates, projected into a three-dimensional framework over a time span of 100 ns. This accumulated data facilitates the clarification of the deterministic motion within each trajectory, understood through orthogonal systems, otherwise referred to as Eigenvectors. The MD simulation trajectory related to the Cα atoms of the protein shows a disorganized arrangement, especially in PC1 and PC2 modes, leaning towards a negative correlation up until the 600th frame. However, the subsequent 400 frames (highlighted in yellow) amalgamate into a more systematic alignment. This convergence of frames into a single cluster signals the cyclical motion demonstrated by the MD trajectories, an outcome of the stable conformational global motion.

## 5 Discussion:

The main cause of mortality for women globally is breast cancer. The disease has gained momentum in recent years and has over taken lung carcinoma in incidence and has turn out to be the most frequent cancer diagnosed globally (DeSantis et al., 2019). Not neglecting the different subtypes and inherent difficulties involved in the aggressiveness and complexity of breast cancer, such as abnormal activation/deregulation of signaling pathways. Furthermore, despite the recent treatment options like surgery, chemotherapy etc. the lethality of breast cancer is alarming (Molnár et al., 2017, Mehraj et al., 2021, Rossing et al., 2021).Moreover, natural compounds are considered a treatment option against breast carcinoma due to their lower negative effects and specificity in targeting important proteins involved in the aberrant activation of pathways in breast carcinoma (Dutt et al., 2017, Varghese et al., 2018, Qayoom et al., 2022). Juglanthraquinone C, a novel compound found in the bark of *Juglans mandshurica* Maxim (Juglandaceae) is a promising compound having cytotoxicity against hepatocellular carcinoma (Yao et al., 2012). However, not much data is available on the molecular mechanisms followed by this compound. Therefore, we aimed to investigate the molecular mechanism followed by Juglanthraquinone C against breast cancer that has not been studied till now and we are the first to undergo this study against breast cancer. To study the molecular mechanisms followed by Juglanthraquinone C in breast cancer, we used a network pharmacology method.

**Fig. 12:**
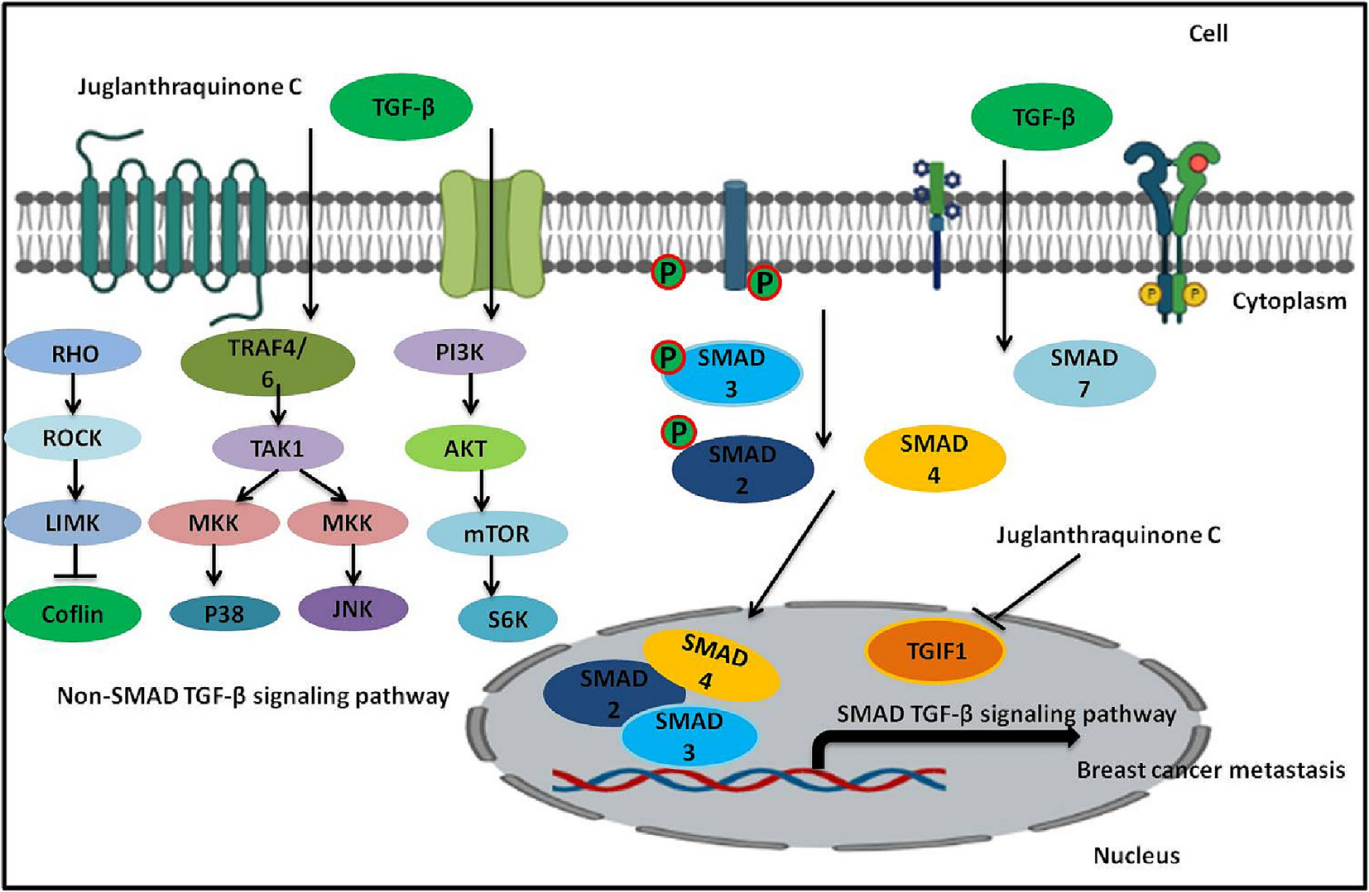
Diagrammatic representation of the involvement of TGF-β signaling pathway in breast cancer metastasis and inhibitory action of Juglanthraquinone C on TGIF1 protein to reduce the breast cancer metastasis.

The network pharmacology analysis suggested that the therapeutic efficacy of Juglanthraquinone C against breast cancer was directly associated with 20 signaling pathways and 31 potential target genes. In the present study, among the top 20 signaling pathways, a hub signaling pathway TGF-β signaling pathway was identified as the most prominent one and was selected for further analysis. TGF-β, a key cytokine has received more attention recently for treating human disorders (Qayoom et al., 2023, Tie et al., 2022). TGF-β is a crucial controller of healthy mammary tissue growth and function as well as the formation and spread of breast tumors (Arteaga et al., 1996). TGF-β is assumed to speed up tumor growth in later stages of the disease, in part by making tumor cells more mobile and invasive (Welch et al., 1990, Oft et al., 1996). Tumor-accelerating actions of TGF-β are linked with amplified TGF-β production by cancer cells during tumor growth (Reiss and Barcellos-Hoff 1997). Recent research has shown that excessive TGF-β supports the epithelial-mesenchymal transition (EMT) and excessive ECM formation, and it also leads to a variety of biochemical anomalies and malfunctions, including fibrosis, immune failure, and cancers (Chakravarthy et al., 2018, Su et al., 2020, Liu et al., 2021, Qayoom et al., 2023. Anti-TGF-β methods have been created to treat fibrosis and cancer because TGF-β plays a significant part in these diseases (Meng et al., 2016). Numerous clinical trials conducted in recent years have established the effectiveness of TGF-β targeted medications in treating a range of tumor and fibrotic illnesses (Mehraj et al., Declèves and Sharma, 2014, Morris et al., 2014, Herbertz et al., 2015). TGF-beta pathway decline in rodents has shown that TGF-beta is a key player in the emergence of cancer. Several investigations have demonstrated that TGF-β regulates the cellular environment and other cytokine impacts, which both contribute to the formation of cancer (David and Massagué 2018, Mehraj et al., 2022, Sofi et al., 2022). Another study discovered that genetic Tgif1 silencing in mice increased KrasG12D-driven PDAC, indicating that TGF-β signaling and TGIF1 may both play multiple roles in carcinogenesis (Parajuli et al., 2019). TGF-β controls cancer cell survival to affect the development of breast carcinoma Fig. 12 (Ehata et al., 2007). Furthermore, because breast carcinoma can rapidly expand to the brain lung, bone, and liver, which is deadly, the function of TGF-β in breast carcinoma metastasis should be highlighted (Medeiros and Allan 2019, Sofi et al., 2022, Sofi et al., 2023, Mir et al., 2020).

In our study, the Kyoto Encyclopedia of Genes and Genomes pathway enrichment analysis of 31 targets suggested that a total of 20 top signaling pathways were involved in breast cancer occurrence and development. This indicated that the effect of phytocompounds acts on multiple pathways for the breast cancer diagnosis. Based on how often each gene appears in the compound-gene network, TP53 showed the highest occurrence of protein interaction, followed by *TP53, JUN, IGF1R, FOS, SMAD3, CDC42, HBEGF, BMP2, TGIF1, AREG*, and other target genes.

Furthermore, an *in-silico* docking analysis of the respective compound Juglanthraquinone C was carried out against the 31 potential targets using AutoDock version 4.2.6 software (Morris et al., 2008). Based on docking scores, the drug had encouraging effectiveness against the TGIF1 protein. Among all the selected targets, Juglanthraquinone C showed greater efficacy towards TGIF1. Juglanthraquinone C interacts well with key amino acid residues and fits comfortably into the TGIF1 protein's active areas (Fig. 9). Juglanthraquinone C was strongly bound to the protein, with the lowly binding energy of −9.9 kcal/mol and exhibited conventional hydrogen bonds formed with Arg66, Asn68 and Asn71 residues and formed pi-sigma and pi-alkyl interactions with Val33 (Fig. 9). Therefore, it can be inferred from the molecular docking studies that Juglanthraquinone C has a high affinity for the protein TGIF1. Additionally, using molecular dynamics models, the stability of the sample TGIF1 and Juglanthraquinone C complex was further investigated. The RMSD plots display consistent patterns in the MD findings throughout the full simulation session (Fig. 10**A and B**). The RMSF plots demonstrate that TGIF1 can accept the drug at the binding pocket with great plasticity. The Rg plots demonstrated that the protein remained compact throughout the experiment. The average findings obtained demonstrated the compactness of the protein backbone. The variations of the residues were looked at in order to comprehend how they behaved during the simulation experiment.

## 6 Conclusion:

In the present study, we explored the prospective mechanisms of Juglanthraquinone C present in *Juglans mandshurica* Maxim (Juglandaceae) in suppressing breast carcinoma by network pharmacology-based analysis included with docking technique and MD simulation studies. Through network pharmacology studies, 20 cancer-related pathways were identified where enzyme linked receptor protein (TGF-β signaling) signaling pathway and regulation of cell death was prominent. The outcome of docking experiments revealed that the compound under study, Juglanthraquinone C demonstrated the highest binding energies to the proteins involved in TGF-β signaling in breast cancer. In conclusion, our study is the first to employ NP-based analysis to methodically examine the potential pharmacological and molecular processes of Juglanthraquinone C in the therapy of breast cancer. According to the results, Juglanthraquinone C, which has a number of targets and pathways, might be an effective treatment for breast cancer. Additionally, our research provides a theoretical foundation for future developments in breast cancer treatment. Since there hasn't been much research on Juglanthraquinone C's ability to prevent breast cancer and because our work was based on data mining and analysis, additional reliable studies are required to back up our conclusion.

## Funding

This work was funded by the JK Science Technology & Innovation council DST India with grant No. JKST&IC/SRE/885–87.

## Declaration of Competing Interest

The authors declare that they have no known competing financial interests or personal relationships that could have appeared to influence the work reported in this paper.
